# Genome-wide immunity studies in the rabbit: transcriptome variations in peripheral blood mononuclear cells after in vitro stimulation by LPS or PMA-Ionomycin

**DOI:** 10.1186/s12864-015-1218-9

**Published:** 2015-01-23

**Authors:** Vincent Jacquier, Jordi Estellé, Barbara Schmaltz-Panneau, Jérôme Lecardonnel, Marco Moroldo, Gaëtan Lemonnier, Jason Turner-Maier, Véronique Duranthon, Isabelle P Oswald, Thierry Gidenne, Claire Rogel-Gaillard

**Affiliations:** INRA, UMR 1313 Génétique Animale et Biologie Intégrative, Domaine de Vilvert, F-78350 Jouy-en-Josas, France; AgroParisTech, UMR 1313 Génétique Animale et Biologie Intégrative, Domaine de Vilvert, F-78350 Jouy-en-Josas, France; INRA, GenPhySE (Génétique, Physiologie et Systèmes d’Elevage), F-31326 Castanet-Tolosan, France; Université de Toulouse, INP, ENSAT, GenPhySE (Génétique, Physiologie et Systèmes d’Elevage), F-31326 Castanet-Tolosan, France; Université de Toulouse, INP, ENVT, GenPhySE (Génétique, Physiologie et Systèmes d’Elevage), F-31076 Toulouse, France; INRA, UMR1198 Biologie du Développement et Reproduction, Domaine de Vilvert, F-78350 Jouy-en-Josas, France; Broad Institute of MIT and Harvard, Cambridge, MA 02142 USA; INRA, UMR1331, Toxalim, Research Center in Food Toxicology, 180 chemin de Tournefeuille, BP 93173, F-31027 Toulouse, France; Université de Toulouse, INP, UMR1331, Toxalim, Research Center in Food Toxicology, F-31000 Toulouse, France

**Keywords:** Rabbit, Transcriptome, Immune response, Microarray, Peripheral blood mononuclear cells, In vitro stimulation, LPS, PMA-Ionomycin

## Abstract

**Background:**

Our purpose was to obtain genome-wide expression data for the rabbit species on the responses of peripheral blood mononuclear cells (PBMCs) after in vitro stimulation by lipopolysaccharide (LPS) or phorbol myristate acetate (PMA) and ionomycin. This transcriptome profiling was carried out using microarrays enriched with immunity-related genes, and annotated with the most recent data available for the rabbit genome.

**Results:**

The LPS affected 15 to 20 times fewer genes than PMA-Ionomycin after both 4 hours (T4) and 24 hours (T24), of in vitro stimulation, in comparison with mock-stimulated PBMCs. LPS induced an inflammatory response as shown by a significant up-regulation of IL12A and CXCL11 at T4, followed by an increased transcription of IL6, IL1B, IL1A, IL36, IL37, TNF, and CCL4 at T24. Surprisingly, we could not find an up-regulation of IL8 either at T4 or at T24, and detected a down-regulation of DEFB1 and BPI at T24. A concerted up-regulation of SAA1, S100A12 and F3 was found upon stimulation by LPS.

PMA-Ionomycin induced a very early expression of Th1, Th2, Treg, and Th17 responses by PBMCs at T4. The Th1 response increased at T24 as shown by the increase of the transcription of IFNG and by contrast to other cytokines which significantly decreased from T4 to T24 (IL2, IL4, IL10, IL13, IL17A, CD69) by comparison to mock-stimulation. The granulocyte-macrophage colony-stimulating factor (CSF2) was by far the most over-expressed gene at both T4 and T24 by comparison to mock-stimulated cells, confirming a major impact of PMA-Ionomycin on cell growth and proliferation. A significant down-regulation of IL16 was observed at T4 and T24, in agreement with a role of IL16 in PBMC apoptosis.

**Conclusions:**

We report new data on the responses of PBMCs to LPS and PMA-Ionomycin in the rabbit species, thus enlarging the set of mammalian species for which such reports exist. The availability of the rabbit genome assembly together with high throughput genomic tools should pave the way for more intense genomic studies for this species, which is known to be a very relevant biomedical model in immunology and physiology.

**Electronic supplementary material:**

The online version of this article (doi:10.1186/s12864-015-1218-9) contains supplementary material, which is available to authorized users.

## Background

The rabbit (*Oryctolagus cuniculus*) is a wild and domesticated species that is used for many purposes including production for meat and fur, biotechnological applications (e.g. antibody production, cloning), and as a tool for the study of nutrition, reproduction, toxicology, pharmaceutical research, and pathologies [1-4]. In fact, the rabbit has been extensively used as a model to study infectious diseases such as tuberculosis, syphilis, anthrax, tularemia, poxvirus diseases, and hepatitis E. Rabbits are also used for research on autoimmune diseases such as systemic lupus erythematosus [5]. The rabbit immune system generates antibody diversity and optimizes affinity through mechanisms that are more efficient than those of mice and other rodents. Due to their high specificity and affinity, rabbit antibodies are commercially available for a large number of target antigens.

The rabbit has been included in the Mammalian Genome Project [6] and a 2X followed by a 7X coverage version of the rabbit genome sequence has been released in 2005 and 2009, respectively. The reference genome assembly OryCun2.0 (AAGW00000000.2, http://www.ncbi.nlm.nih.gov/genome/?term=oryctolagus%20cuniculus) has provided insights to decipher the impact of the domestication process on shaping genomes [7]. Genomic tools are growing for the rabbit species, complementary to the onset of more and more cost-effective high throughput sequencing methodologies, paving the way to large scale genome-wide expression studies [4]. However, very few genome-wide expression studies have been reported to date in the rabbit and most of which have focused on early embryogenesis [8-10] pluripotent stem cells [11], implantation during gestation [12], ocular research [13,14] and response of different organs to Eimeria infections [15-17]. Although rabbits have proven very useful in immunological and infectious disease research, genome-wide expression studies targeting the immune response to various challenges are still limited. Our aim was to create genome-wide gene expression datasets representative of various in vitro stimulations of immune cells in rabbits in order to increase knowledge on the rabbit immune responses, to provide new rabbit-specific data for comparative immunology, and to promote that transcriptomics-based approaches are efficient to provide molecular phenotype information to analyze immunity in the rabbit species. We chose to study variations of the transcriptome in rabbit peripheral blood mononuclear cells (PBMCs) using two different stimulations expected to induce either an innate immune response (lipopolysaccharide (LPS stimulation)) or a lymphocyte proliferation (mixture of phorbol myristate acetate (PMA) and ionomycin (PMA-Ionomycin stimulation)). LPS is part of the outermost layer of gram-negative bacteria and is a pathogen-associated molecular pattern (PAMP) used for in vitro studies of the innate immune response after bacterial infection. PMA, in conjunction with ionomycin, act preferentially on growth and proliferation of immune cells, and stimulate the intracellular production of the cytokines IL2 and IL4. Both stimulations were chosen as they are classically used to induce distinct types of immune responses for further characterization. This study stands as the rabbit counterpart of a similar study that was conducted in pig [18], and that has provided much new data on the main gene pathways that were modified following each stimulation. For this purpose, we have designed a rabbit long oligonucleotide-based DNA microarray, by enriching a previously customized gene expression design (GPL16482) with a set of well-annotated genes known to be involved in immune and inflammatory responses. We report the striking features of gene expression changes in response to both stimulations over time, relying on gene expression microarrays that are informative, cost-effective and easy to use for genome-wide expression studies in the rabbit. To our knowledge, our study reports for the first time the modifications of transcriptome profiling of PBMCs upon LPS of PMA-Ionomycin stimulation in the rabbit species.

## Results

### A well-annotated gene expression platform enriched in immunity-related genes for the rabbit species

The rabbit gene expression microarray used in this study combines the customized microarray design (GPL16482) with a set of 453 specific rabbit genes involved in the immune system process (GO: 0002376), and identified to be missing on the previous array. Gene set information is available in the Additional file 1. This custom microarray has been updated with the latest annotation generated by the rabbit sequencing consortium [7].

The resulting microarray platform includes 61655 probes, 50443 of which have chromosome coordinates (82%) and 44670 are annotated (72%). These annotated probes specify 11899 genes with a symbol approved by the HUGO Gene Nomenclature Committee. Indeed, the majority of probes found differentially expressed (DE) after LPS or PMA-Ionomycin treatment were annotated, as shown on Table 1.

**Table 1:** **Number of genes found up- or down-regulated (FDR < 0.05) after stimulation, and percentage of probe annotation**

**Stimulation**	**Genes up-regulated**	**Genes down-regulated**	**Total DE genes**	**Percentage of annotation**
LPS at T4	35	281	316	90.82
LPS at T24	212	165	377	90.98
PMA-Ionomycin at T4	2273	2477	4750	80.93
PMA-Ionomycin at T24	3414	4039	7453	75.51

### Clustering of samples and comparative effect of LPS and PMA-Ionomycin stimulations

**Figure 1:**
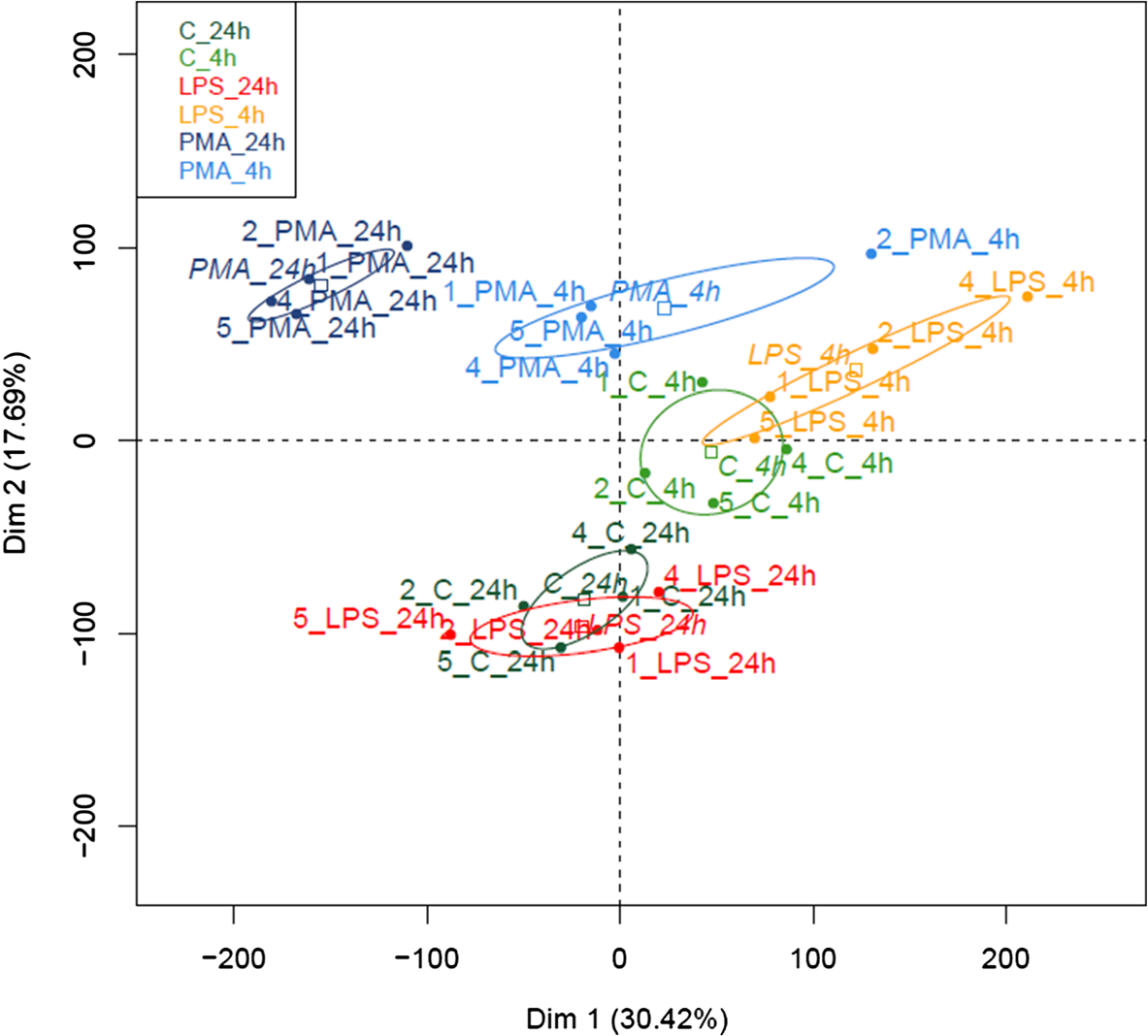
**Principal component analysis of microarray data, classified by biological replicates.**

PBMCs were isolated from four rabbit blood samples and either mock-stimulated or stimulated with LPS or PMA-Ionomycin for 4 (T4) and 24 (T24) hours. The principal component analysis (PCA; Figure 1) revealed that all samples stimulated by PMA-Ionomycin clustered according to the treatment and the time of stimulation (T4 or T24). The two time-based clusters were clearly separated from mock-stimulated or LPS-stimulated samples. Conversely, mock-stimulated and LPS-stimulated samples defined two close clusters at T4 but a unique cluster that consisted of samples from both treatments at T24. The hierarchical clustering analysis (HCA; Additional file 2) confirmed the PCA results. Since no sample was found as an outlier, all biological samples were considered for the following steps of the analysis.

The differential expression analysis revealed that the number of DE genes increased with time for both stimulations (Table 1). However, the number of DE genes after LPS stimulation at T4 and T24 was approximately 15 to 20 times lower than after PMA-Ionomycin stimulation. Sets of 316 and 377 DE genes were found after LPS stimulation at T4 and T24, respectively (FDR < 0.05). By contrast, sets of 4750 and 7453 DE genes were detected at T4 and T24, respectively, after PMA-Ionomycin stimulation (FDR < 0.05). In addition, the fold change (FC) values with LPS-stimulation varied from −3.38 to 3.20 at T4 and from −9.64 to 50.10 at T24, compared to mock-stimulation. For PMA-Ionomycin stimulation, the FC values ranged from −48.36 to 227.98 at T4 and from −146.61 to 57.58 at T24 (Table 2). The differential analysis showed that the transcriptome profile of PBMCs varied considerably after PMA-Ionomycin treatment in comparison to the LPS treatment. The striking differences in the number of DE genes and the FC values between LPS and PMA-Ionomycin treatments might explain the close clustering between LPS and mock-stimulated samples.

**Table 2:** **Top-ten DE genes after LPS or PMA-Ionomycin stimulation, T4 and T24**

		**T4**			**T24**		
		**Gene symbol**	**FC** ^**1**^	**Adj.P.Val**	**Gene symbol**	**FC** ^**1**^	**Adj.P.Val**
LPS	Up - regulation	CXCL11	3.20	4.11E-02	F3	50.10	2.91E-04
		LMTK2	2.69	1.45E-02	IL6	26.96	2.09E-04
		RETN	2.35	3.11E-02	SAA1	26.27	9.37E-04
		IL12A	2.29	3.85E-02	IL1B	18.12	2.96E-03
		HENMT1	2.28	4.87E-02	IL10	17.61	6.48E-04
		UBE2D2	2.11	4.44E-02	IL36G	17.11	9.49E-05
		EVA1B	2.10	4.96E-02	S100A12	15.74	4.69E-02
		SPTBN4	1.95	3.85E-02	TNIP3	11.33	1.57E-03
		PKHD1L1	1.56	4.11E-02	HBEGF	9.67	2.49E-05
		NOS2	1.47	4.72E-02	CCL4	9.10	4.12E-02
	Down - regulation	CTSE^2^	−3.38	3.11E-02	PALLD	−9.64	2.33E-05
		CHI3L1	−2.90	3.44E-02	MLANA	−8.12	4.55E-07
		SAMHD1	−2.90	3.85E-02	DEFB1	−6.9	3.05E-03
		CLEC4D	−2.89	3.28E-02	ATP6V0D2	−6.59	4.25E-06
		ALDOA	−2.83	1.92E-02	CLEC7A	−6.23	6.12E-06
		TKT	−2.82	2.25E-02	SLAMF7	−6.09	1.86E-04
		ATP6V0B	−2.79	1.25E-02	ASPA	−5.15	3.65E-04
		PGAM1	−2.78	3.84E-02	CTBS	−4.98	3.24E-04
		COMT	−2.78	6.21E-03	BPI	−4.92	5.73E-03
		FHOD1	−2.78	1.63E-02	MPEG1	−4.46	1.65E-03
PMA - Ionomycin	Up - regulation	CSF2^3^	227.98	5.78E-12	CSF2^3^	57.58	1.99E-10
		IL13^3^	144.98	4.40E-18	BCO2^4^	51.95	1.07E-13
		IL4^3^	105.19	7.22E-12	CD70^4^	34.74	2.65E-11
		NFKBID^3^	61.72	3.67E-14	RGCC^3^	31.77	4.15E-12
		TBX21^3^	57.61	1.40E-14	SPRY1^3^	29.99	6.87E-17
		SPRY1^3^	53.40	2.41E-18	ZBTB32^3^	29.31	1.26E-11
		IL2^3^	40.01	9.61E-18	CCNB1^4^	21.20	3.78E-11
		RGCC^3^	33.61	5.83E-12	I2^3^	20.36	1.07E-15
		MMP3^3^	33.33	3.67E-14	LAG3^3^	19.70	7.93E-12
		IL10^3^	33.33	2.28E-06	NCAPG^4^	18.80	2.07E-12
	Down - regulation	FAM26F	−48.36	3.10E-11	S100A8^3^	−146.61	4.01E-08
		RBP4^3^	−32.07	2.32E-08	RBP4^3^	−67.53	4.27E-10
		TRAF3IP3^3^	−21.39	6.70E-11	MGP	−58.62	4.98E-13
		IL16^3^	−18.62	4.17E-10	DEFB1	−51.43	6.61E-09
		LTB^3^	−18.29	1.24E-07	S100A12^3^	−42.78	1.89E-04
		TM4SF5^3^	−16.79	1.27E-09	ACTA2	−40.95	1.71E-12
		CD79B	−16.07	1.37E-06	LTB^3^	−33.98	2.45E-09
		MYRFL^3^	−15.90	3.67E-14	PID1^3^	−33.27	7.21E-09
		TLR10	−15.62	7.66E-11	TM4SF5^3^	−32.16	2.12E-11
		TNFAIP8L2^3^	−14.95	6.25E-05	CYBB^3^	−31.07	8.87E-12

^1^FC: fold change.

^2^Genes found to be DE in the same direction (either down- or up-regulated compared to mock-stimulation) at both T4 and T24 post-stimulation by LPS.

^3^Genes found to be DE in the same direction (either down- or up-regulated compared to mock-stimulation) at both T4 and T24 post-stimulation by PMA-Ionomycin.

^4^Genes that were down-regulated at T4 in contrast to T24.

### Genes and biological functions affected by LPS stimulation

**Figure 2:**
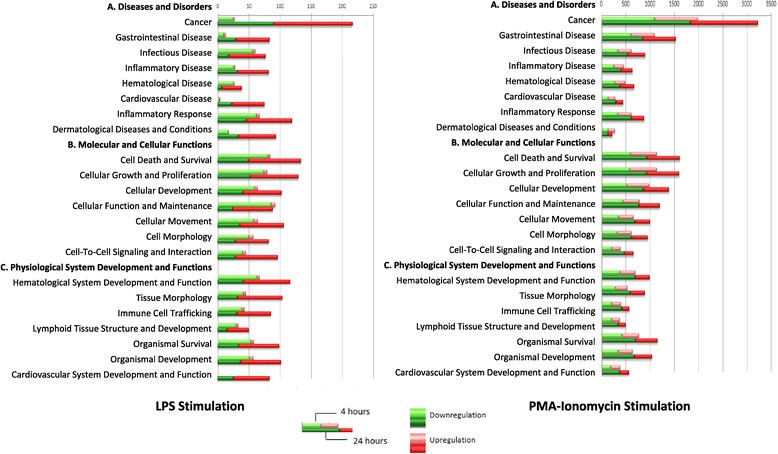
**Top biological functions classified by the IPA system, with corresponding number of molecules after LPS and PMA-Ionomycin activation.**

At T4, most DE genes were down-regulated (89% of DE genes) as shown in Table 1 and Figure 2. The most down-regulated gene was the Cathepsin E gene (CTSE), which is an aspartyl protease, followed by the chitinase 3-like 1 gene (CHI3L1), which encodes a glycoprotein. Other genes of interest that were down-regulated include the SAM domain and HD domain 1 gene (SAMHD1), the C-type lectin domain family 4, member D gene (CLEC4D), which encodes a member of the C-type lectin/C-type lectin-like domain (CTL/CTLD) superfamily, and the aldolase A, fructose-bisphosphate gene (ALDOA). The top ten down-regulated genes at T4 are listed in Table 2. The CD14 molecule gene, known to encode a surface antigen expressed on monocytes and macrophages and involved in mediating the innate immune response to LPS, was found to be down-regulated with a FC value of −2.31 (Additional file 3). The most up-regulated genes were the chemokine (C-X-C motif) ligand 11 (CXCL11), the lemur tyrosine kinase 2 (LMTK2), which is involved in endosomal membrane trafficking, and the resistin gene (RETN). Other genes that were up-regulated include the interleukin 12A (IL12A) together with the nitric oxide synthase 2, inducible gene (NOS2) which is inducible by LPS in the presence of IL12, and the ubiquitin-conjugating enzyme E2D 2 (UBE2D2).

At T24, the LPS activation revealed a switch towards a majority of genes being up-regulated (212 up vs. 165 down-regulated genes; see Table 1 and Figure 2), with higher FC values than at T4, ranging from −9.64 to 50.10 (Table 2). The most up-regulated gene was the coagulation factor III (thromboplastin, tissue factor) (F3). Among the top ten up-regulated genes (Table 2), an important subset of genes was connected to the inflammation process, and included genes that encode the proinflammatory cytokines IL6, IL1B, and IL36G, the Serum Amyloid A1 protein (SAA1), the chemokine (C-C motif) ligand 4 (CCL4), and the interleukin 10 (IL10) known to have pleiotropic effects on immunoregulation and inflammation. TNF was also found to be up-regulated (Additional file 4) together with the gene encoding the TNFAIP3 Interacting Protein 3 (TNIP3). In addition, the heparin-binding EGF-like growth factor (HBEGF) and the S100 calcium binding protein A12 (S100A12) were in the top ten up-regulated genes. The HBEGF gene has been reported to encode a cell surface binding protein potentially involved in macrophage-mediated cellular proliferation, and the S100A12 gene has been shown to be involved in cell cycle progression and differentiation. The most down-regulated gene was the Palladin, Cytoskeletal Associated Protein gene (PALLD). The top-ten down-regulated genes (Table 2) also included the bactericidal/permeability-increasing protein gene (BPI) and the defensin beta 1 gene (DEFB1). BPI encodes a LPS binding protein and has bactericidal activity on gram-negative organisms. DEFB1 belongs to the defensins reported to form a family of microbicidal and cytotoxic peptides made by neutrophils, and encodes an antimicrobial peptide involved in the resistance of epithelial surfaces to microbial colonization.

**Figure 3:**
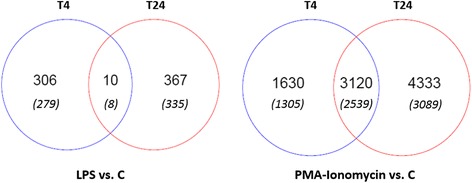
**Venn diagram of probes differentially expressed after LPS or PMA-Ionomycin stimulation at 4 and 24 hours.** The value between parentheses indicates the number of well-annotated genes.

A very limited set of 10 genes corresponding to eight annotated genes mostly involved in cellular metabolism was DE at both T4 and T24 following LPS stimulation (Figure 3). Four genes were down-regulated at both times (Table 3), including the cathepsin E (CTSE), which encodes an asparatyl protease, the acid phosphatase 5, tartrate resistant gene (ACP5), the lamin A/C gene (LMNA), and the interferon regulatory factor 5 (IRF5). Five probes specifying three genes were found to be DE in opposite orientations at T4 (down-regulation) and T24 (up-regulation). These genes are the membrane protein, palmitoylated 1, 55 kDa gene (MPP1) that encodes a palmitoylated membrane phosphoprotein, the member RAS oncogene family gene (RAB5C), that is a small GTPase, the serologically defined colon cancer antigen 8 (SDCCAG8), and the putative phytanoyl_CoA 2-hydroxylase (PHYH).

**Table 3:** **List of the 10 genes found DE at T4 and T24 upon LPS stimulation**

	**T4**		**T24**	
**Gene name**	**FC** ^**1**^	**Adj.P.Val**	**FC** ^**1**^	**Adj.P.Val**
CTSE	−3.38	3.11E-02	−2.81	3.88E-02
ACP5	−2.17	2.98E-02	−1.87	4.57E-02
LMNA	−2.16	3.85E-02	−2.08	3.51E-02
IRF5	−1.98	4.87E-02	−2.23	1.36E-02
lcaw0026.b.03.a_5.1.fwd2^4^	1.97	1.45E-02	2.12	5.87E-04
MPP1	−2.41	3.85E-02	2.19	4.85E-02
ENSOCUT00000015242^2^	−2.00	1.92E-02	2.24	8.45E-04
lcaw0079.e.09.a_5.1.fwd1^3^	−1.82	1.92E-02	1.83	3.26E-03
RAB5C	−1.80	3.85E-02	1.72	3.51E-02
SDCCAG8	−1.32	3.11E-02	1.29	2.20E-02

^1^FC: fold change.

^2^Putative annotation following BLAST search for sequence similarities: *Oryctolagus cuniculus* phytanoyl-CoA 2-hydroxylase (NCBI Accession number: LOC100338511, info on BLAST score).

^3^Putative annotation following BLAST search for sequence similarities: *Oryctolagus cuniculus* phytanoyl-CoA 2-hydroxylase (NCBI Accession number: LOC100344679; info on BLAST score).

^4^No available annotation.

For the global analysis of biological functions affected by LPS stimulation, the DE gene lists were submitted to the Ingenuity Pathway Analysis (IPA) system. From this analysis 262 (82.9% of DE genes) and 322 (85.4% of DE genes) genes were identified by IPA at T4 and T24, respectively. The global picture provided by Figure 2 highlights that most genes were found down-regulated at T4 conversely to T24. The foremost over-represented down-regulated biological functions (P < 0.05) at T4 were related to Molecular and Cellular Functions (Cellular Function and Maintenance, Cell Death and Survival, Cellular Growth and Proliferation). However, a number of genes involved in biological functions related to inflammatory disease or response were repressed at T4. By contrast, at T24, there was a significant enrichment of up-regulated genes associated with biological functions related to Diseases and Disorders such as inflammatory response, Inflammatory Disease and Infectious Disease. For the Molecular and Cellular Functions, the most represented functions were Cell Death and Survival, Cellular Growth and Proliferation, Cellular Movement, Cellular Development and Cell-to-Cell Signaling and Interactions. For the Physiological System Development and Functions, the most represented function was the Hematological System Development and Function.

**Figure 4:**
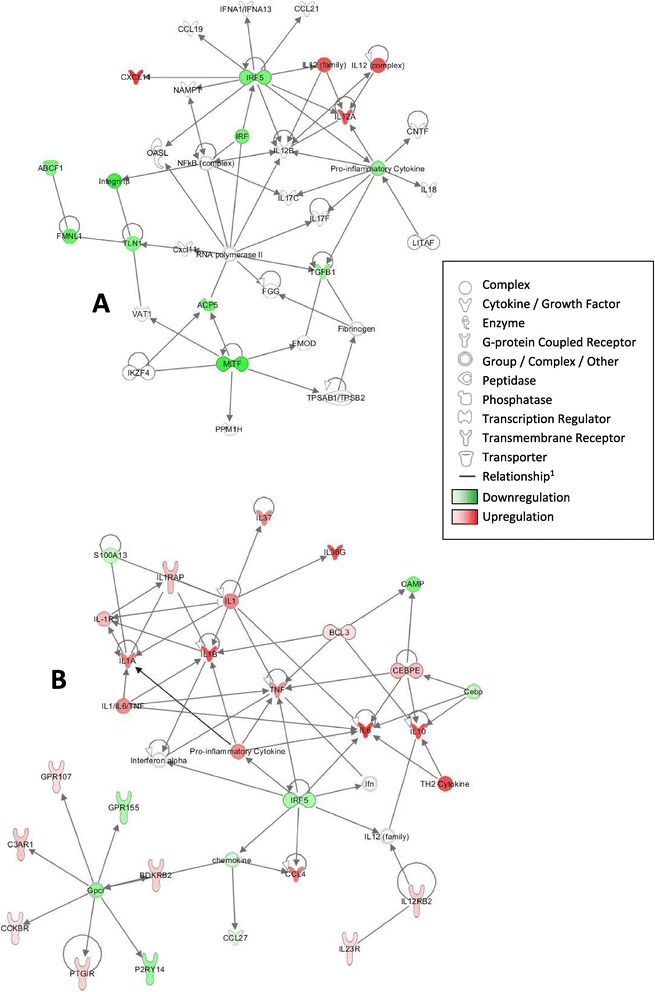
**LPS-related gene networks identified by the IPA system: network at T4 (A) and network at T24 (B).** ^1^All relationships are direct interactions.

The IPA system identified 17 and 21 gene networks at T4 and T24, respectively. The gene network that groups cytokines and chemokines known to be involved in inflammation responses is presented in Figure 4 and draws a picture of the kinetics of inflammatory response to LPS stimulation. At T4, the LPS-related network shown in Figure 4A is involved in Inflammatory Response, Cell Death and Survival, and Cellular Movement. This network grouped 9 DE genes, which included two connected genes, CXCL11 and IL12A, listed in the top-ten up-regulated genes (Figure 4A). They are reported to be directly under the control of IRF5 by IPA.

At T24, the LPS-related network shows the interactions of 24 DE genes (Figure 4B), and provides a picture of the intensification of the inflammatory response together with an increase in the number of up-regulated molecules involved in this process (IL1B, IL6, IL36G, IL37, and CCL4). In addition, a cell differentiation toward a Th2 phenotype is provided by this gene network (GATA3 gene linked to Th2 cytokine cluster and IL10). The variation in cytokine profiling upon stimulation by LPS with time is summarized in Table 4. Unexpectedly, CXCL8 (also known as IL8) was not found to be up-regulated in our experimental conditions.

**Table 4:** **Transcription profile of cytokines in PMBCs stimulated by LPS at T4 and T24**

**Condition**	**ID**	**FC** ^**1**^	**adj.P.Val**
LPS T4	CXCL11	3.20	4.11E-02
	IL12A	2.29	3.85E-02
	NOS2	1.47	4.72E-02
	CD14^2^	−2.31	4.52E-02
LPS T24	IL6^2^	26.96	2.09E-04
	IL1B^2^	18.12	2.96E-03
	IL10^2^	17.61	6.48E-04
	IL36G	17.11	9.49E-05
	IL1A	9.01	2.20E-02
	IL37	7.02	4.16E-05
	TNF	5.92	1.18E-02
	IL1RAP	2.82	2.26E-02
	IL12RB2	2.23	1.14E-03
	IL23R	1.76	1.69E-03
	CCL27	−1.42	3.49E-02

^1^FC: fold change.

^2^Genes used for qRT-PCR validation.

### Genes and biological functions affected by PMA-Ionomycin stimulation

Two large sets of genes were found DE at T4 and T24 (Table 1), with a slight tendency toward more up-regulated genes than down-regulated genes at both times (2273 down- versus 2477 up-regulated probes at T4; 3414 down- versus 4039 up-regulated probes at T24). As shown in Table 2 (and Additional files 5 (T4) and Additional file 6 (T24)), there was a decrease over time in the FC values for up-regulated genes (maximum FC was 227.98 and 57.58 at T4 and T24, respectively) but an increase over time in the FC of down-regulated genes (minimum FC was −48.36 and −146.61 at T4 and T24, respectively). A set of 3120 DE probes was shared at both times, and could be divided into a subset of 2378 probes that were continually DE in the same directions at T4 and T24 (1091 probes down-regulated and 1287 probes up-regulated), a subset of 515 probes that were down-regulated at T4 and up-regulated at T24, and a third subset of 227 probes that were up-regulated at T4 but down-regulated at T24.

The genes CSF2, IL2, and RGCC (gene regulator of cell cycle) were among the top ten genes found over-expressed at T4 and T24. The granulocyte-macrophage colony-stimulating factor (CSF2) was by far the most over-expressed gene with a positive FC of 227.98 at T4 and 57.58 at T24 (Table 2). CSF2 encodes a cytokine that controls the production, differentiation, and function of granulocytes and macrophages, and the RGCC gene encodes a protein thought to regulate cell cycle progression. All top ten over-expressed genes at T4 were still up-regulated at T24. Conversely, the genes BCO2, CD70, CCNB1, and NCAPG found in the top ten over-expressed genes at T24 were found to be down-regulated at T4, showing a switch in gene regulation between T4 and T24. The beta-carotene oxygenase 2 gene (BCO2) encodes an enzyme, which oxidizes beta-carotene during the biosynthesis of vitamin A. CD70 molecule gene (CD70) encodes a cytokine that belongs to the TNF ligand family. CD70 is a ligand for TNFRSF27/CD27 and a surface antigen on activated B and T lymphocytes, and contributes to T cell activation. The cyclin B1 gene (CCNB1) encodes a regulatory protein involved in mitosis. The non-SMC condensin I complex, subunit G gene (NCAPG) encodes a subunit of the condensin complex, which is responsible for the condensation and stabilization of chromosomes during mitosis and meiosis.

The genes LTB and FPR1 were both found in the top ten most repressed genes at T4 and T24 post stimulation. The lymphotoxin beta (TNF superfamily, member 3) (LTB) encodes a protein which is an inducer of the inflammatory response system and is involved in normal development of lymphoid tissue. In addition, there was an intensification of the repression of the two genes S100A8 (S100 calcium binding protein A8) and S100A12 (S100 calcium binding protein A12), which encode proteins of the S100 family containing 2 EF-hand calcium-binding motifs, involved in cell cycle progression and differentiation. The defensin beta 1 (DEFB1) gene was repressed only at T24.

The cytokine and chemokine profiles revealed a continued up-regulation over time of CSF2, IL2, IL4, IL10, IL13, IL17A, IL31, IL9, IFNG, CCL1, CCL2, and CCL20 (Table 5), and a down-regulation of IL16, IL37, IL36G, and IL15. The CD69 molecule gene (CD69), known to be induced by the activation of T lymphocytes, was found to be over-expressed at T4 (FC = 12.1) and T24 (FC = 5.07). At T4, there was a notable opposite regulation between over-expressed IL13, IL4, IL2, and IL10, and repressed IL16 and LTB. This opposite regulation was maintained at T24. IL13 is an immunoregulatory cytokine primarily produced by activated Th2 cells reported to inhibit the production of pro-inflammatory cytokines and chemokines. IL16 is a pleiotropic cytokine that functions as a chemoattractant and a modulator of T cell activation. At T4, a specific up-regulation of IL17F, CXCR7, IL21, IL22, IL33, and TNFRSF21, together with a repression of IL18 and CXCL10 were detected. At T24, a specific up-regulation of CCL4, CCL22, CCL27, IL34, TNF, IL12A, and IL6, together with a repression of IL1R2, CXCR4, IL1B, TNFSF13, and IL1RAP were shown.

**Table 5:** **Transcription profiles of cytokines and transcription factors in PMBCs stimulated by PMA-Ionomycin at T4 and T24**

**ID**	**FC** ^**1**^		**ID**	**FC** ^**1**^	**ID**	**FC** ^**1**^
	**T4**	**T24**		**T4_specific**		**T24_specific**
CSF2	227.98	57.58	IL17F	7.80	CCL4^2^	5.02
IL13	144.98	4.68	CXCR7	5.35	CCL22	2.99
IL4	105.19	18.23	IL21	4.75	CCL27	1.99
IL2^2^	40.01	20.36	IL22	4.50	IL34	5.91
IL10^2^	33.33	3.46	IL33	2.33	TNF^2^	5.15
IL17A	19.34	4.27	TNFRSF21	1.65	IL12A	4.03
CD69	12.11	5.08	IL17RE	−1.37	IL6^2^	4.03
IFNG^2^	8.50	12.31	ILDR2	−1.68	ILF3	2.24
CCL2	6.95	5.06	CXCL9	−1.71	TNFRSF25	1.78
TNFRSF12A	6.90	3.53	IL12RB2	−1.77	TNFRSF13B	1.62
CCL1	5.40	13.78	IL18	−1.91	TNFRSF8	1.60
TNFSF14	5.07	2.49	IL18R1	−1.92	ILDR1	−1.18
CCL20	4.28	5.05	IFNAR1	−1.94	CCL14	−1.32
IL31	3.05	1.88	IFNGR1	−2.12	IL1RAPL2	−1.35
IL6ST	2.93	−2.44	TNFSF10	−2.48	IL27RA	−1.43
TNFRSF9	2.87	2.71	IL18RAP	−2.64	IL18BP	−1.68
ILF2	2.26	2.04	IFNAR2	−2.71	IL22RA2	−1.77
IL9	1.90	1.95	IFNGR2	−2.79	CCL21	−1.83
TNFSF11	1.46	2.12	CXCL10	−6.13	IL1RN	−2.04
IL6R	−1.77	−1.52			TNFRSF11B	−2.06
TNFSF8	−1.90	−3.19			TNFRSF1B	−2.13
IL3RA	−1.95	−5.05			IL7R	−2.57
IL10RA	−2.08	−2.15			TNFAIP3	−2.61
IL1RL1	−2.18	−2.66			IL13RA1	−2.86
TNFRSF17	−2.28	−1.64			TNFAIP8L3	−2.97
TNFRSF1A	−2.35	−3.27			IL2RG	−4.09
IL15	−2.46	−2.91			IL1RAP	−6.58
IL17RA	−2.54	−4.57			TNFSF13	−8.80
IL36G	−4.84	−2.97			IL1B^2^	−11.68
IL37	−5.31	−2.17			CXCR4	−14.72
TNFAIP8L2	−13.00	−9.72			IL1R2	−16.48
IL16	−18.62	−7.92			STAT4	9.67
TBX21	57.61	12.01			STAT1	1.89
STAT5A	3.41	1.70			GATAD2B	−1.51
STAT5B	3.14	2.09			GATA2	−1.64
GATA1	−1.45	−2.12			THBS1	−4.75

^1^FC: fold change.

^2^Genes used for qRT-PCR validation.

The transcription factors TBX21, STAT5A, and STAT5B were up-regulated at T4 and T24, whereas GATA1 was slightly repressed at both times (Table 5). TBX21 is the T-box 21 gene that stands as the human ortholog of the mouse Tbx21/Tbet gene. This gene was reported to act as a Th1 cell-specific transcription factor and controls the expression of IFNG [19]. STAT5A and STAT5B are both members of the signal transducer and activator of transcription (STAT) family of transcription factors that respond to cytokines and growth factors. STAT5A encodes a protein that is activated by IL2 and CSF2, and STAT5B encodes a protein reported to mediate the signal transduction triggered by various cell ligands, such as IL2 and IL4. At T24, two additional members of the STAT family of transcription factors, STAT4 and STAT1, were found up-regulated and two other members of the GATA families were slightly repressed (GATA2 and GATAD2B). FOXP3 was not found affected by the PMA-Ionomycin stimulation. We checked the differential expression of the thrombospondin gene (THBS1) that was found to be the most repressed gene in porcine PBMCs stimulated for 24 hours by PMA-Ionomycin [18]. In rabbit PBMCs, the THBS1 gene was repressed only at T24 and with a limited FC of −4.75.

The genes encoding class II molecules of the major histocompatibility complex (MHC) were found to be repressed. The most repressed genes belong to the DR series followed by DM, DQ and DP. This repression was initiated early after stimulation since DMA, DMB and DRB1 were already found down-regulated at T4 (Additional file 7). In addition, few probes corresponding to MHC class I transcripts were found down-regulated. Interestingly, and in agreement with data obtained in pig [18], we observed an up-regulation of genes encoding heat shock proteins (HSPs) and many genes involved in the cascade of peptide processing before loading to the MHC molecule binding groove (components of the proteasome (PSMB gene series), calnexin (CANX), tapasin (TAP1)), despite an overall down-regulation of genes encoding class I and II molecules (Additional file 7).

At T4 and T24, subsets of 3575 (75.3%) and 5153 (69.1%) DE genes were mapped into the IPA system. The relative representation of biological functions between T4 and T24 was quite similar (Figure 2). By excluding all genes associated to Cancer, IPA revealed an enrichment of genes principally related to Molecular and Cellular Functions (cell death and survival, cellular growth and proliferation, cellular development, cellular function and maintenance), followed by an enrichment of genes associated with Diseases and Disorders (gastrointestinal disease, infectious disease, inflammatory response), and Physiological System Development and Function (organismal survival, organismal development, hematological system development and function). These observations were reinforced by the analysis of the set of 3123 genes found to be DE at both T4 and T24, and showed a predominance of genes associated with Molecular and Cellular Functions. The 515 genes found down-regulated at T4 and up-regulated at T24 were predominantly associated to the biological functions Cell Cycle, DNA Replication Recombination and Repair, and Cellular Assembly and Organization. For the 227 genes found up-regulated at T4 and down-regulated at T24, there was an enrichment in biological functions relating to Cell Death and Survival, Cell Growth and Proliferation, Cell Development, Tissue Morphology, and Hematological System Development and Function. For these two gene sets, the enriched biological functions aligned well with those found for the genes that revealed no inverted regulation compared to mock-stimulation at T4 and T24.

**Figure 5:**
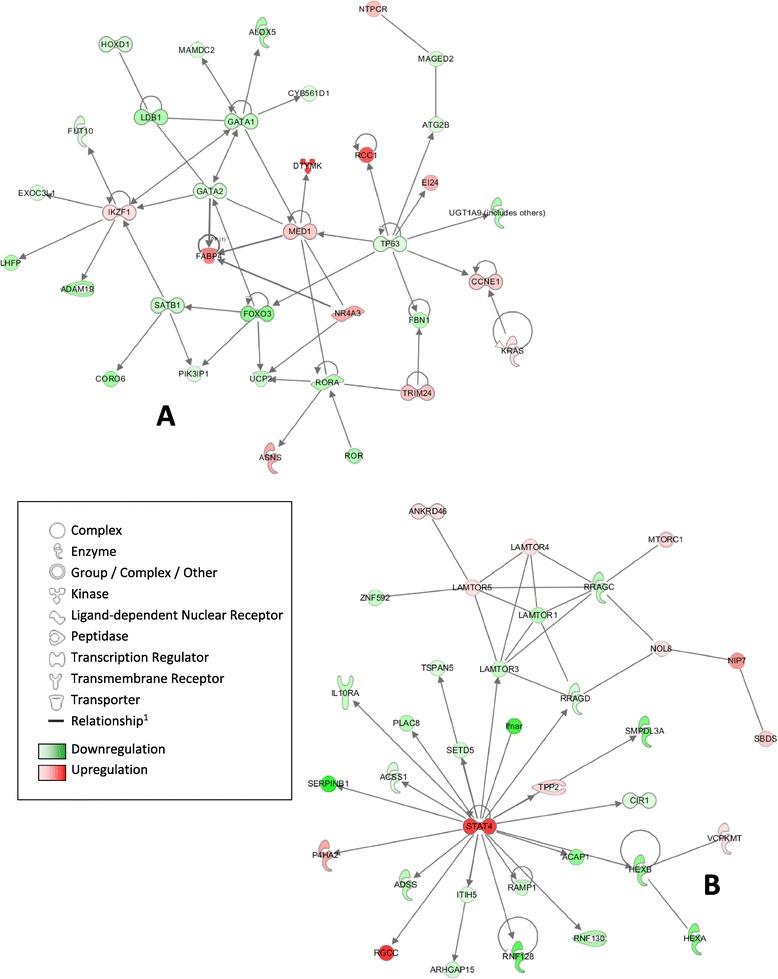
**PMA-Ionomycin-related gene networks identified by the IPA system at T24 (A and B).** ^1^All relationships are direct interactions.

The PMA-Ionomycin network analysis was deliberately limited to 25 networks, including up to 25 genes each. The network shown in Figure 5A highlights transcription factors GATA1 and GATA2, down-regulated at T24 and involved in many aspects of cell growth. In the second network (Figure 5B), STAT4 is up-regulated and in a central position, inhibiting IL10RA but activating RGCC. This transcription factor STAT4 is required for the development of Th1 cells from naïve CD4+ T cells and IFNG production in response to IL12.

### Validation of differentially expressed genes by qRT-PCR

**Figure 6:**
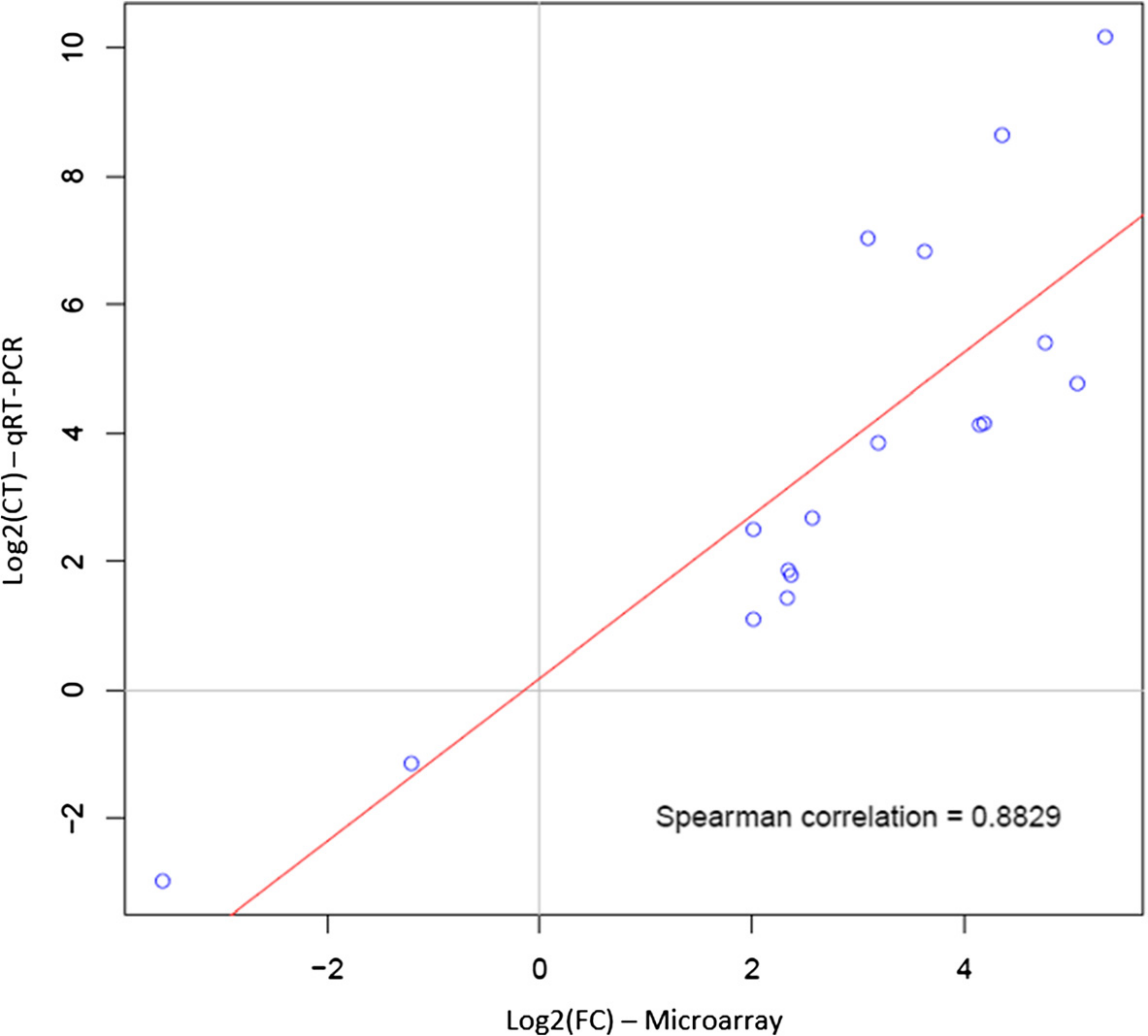
**Comparison between microarray-based (Log2(CT)) and qRT-PCR-based results (Log2(FC)) by Spearman correlation scattered plots.**

A set of eight genes was chosen for microarray data validation by real time quantitative reverse transcription PCR (qRT-PCR). These genes were selected according to their expression level (up- or down-regulated), in order to test genes found DE at T4 and T24 for both LPS and PMA-Ionomycin stimulations (Additional file 8). The B2M and GAPDH genes were included as reference genes for data normalization. A linear regression was performed between the log2(fold change) values of microarray and qRT-PCR data. A significant Spearman correlation coefficient (R^2^ = 0.8829) was calculated between the two techniques (Figure 6), thus validating the microarray data and confirming the reliability of the transcriptome analyses.

## Discussion

### Cytokine profile induced by LPS stimulation in rabbit PBMCs

LPS is an outer membrane component of Gram-negative bacteria and the crystal structure of the trans-envelope protein complex inserting LPS in the outer leaflet of the outer envelope has been very recently described [20,21]. Lipid A is the main PAMP of LPS. Interactions between LPS and TLR4, a sensor for LPS [22] at the surface of macrophages and dendritic cells, trigger the expression of proinflammatory cytokines and activate the innate immune system by MyD88 (Myeloid differentiation primary response gene) dependent or MyD88 independent pathways [23]. In our study, an early up-regulation of IL12A and CXCL11 together with a slight repression of CD14 was found at T4. This LPS-induced CD14 repression observed at T4 was comparable to *in vivo* human studies [24], highlighting the immunodepressive effect of LPS on rabbit PBMCs. Up-regulation of a wider range of inflammatory cytokines was observed at T24 with a differential expression between mock-stimulation and LPS-stimulation for IL6, IL1A, IL1B, and TNF. In LPS-stimulated human whole blood [25], it has been reported that gene activations of both IL6 and TNF were induced early, after only 2–4 hours and then rapidly declined. Several studies have shown the synergy between IL1B and TNF [26,27]. When both cytokines were administered to healthy humans, they induced fever, inflammation, tissue destruction, or resulted in the development of sickness behavior, and depressed mood when combined with IL6 [28,29]. Moreover, IL1B and TNF have been shown to stimulate the expression of IL36G in keratinocytes [30,31], confirming results at T24. Our results suggest a significant triggering of the MyD88 dependent pathway in dendritic cells that is characterized by IL12 production and followed by a T cell differentiation toward Th1 phenotype [32,33], as represented in Figure 5A. The onset of the inflammation process was detected at T4 but clearly amplified at T24 as represented in Figure 5B, showing that the data obtained at T4 did not provide an accurate overall picture of gene expression picture upon LPS stimulation. In addition, gene transcription levels did not return to basal levels at T24, as already shown in pigs [18]. This late increase in inflammatory cytokine transcription contrasts with results on bovine PBMCs for which the inflammation process was triggered much earlier with a maximal expression of inflammation gene expression at T4 [34]. For both T4 and T24 after LPS activation, the interferon regulatory factor 5 (IRF5) was found to be under-expressed. In 2011, Krausgruber et al. [35] highlighted a critical role for the transcription factor IRF5 in human M1 macrophage polarization and defined it as a transcriptional repressor. Moreover, an activation of the Th1 response by IRF5 has been reported.

### Putative specific features of rabbit PBMCs upon LPS stimulation

IL8 (CXCL8 in human) was not DE in our experimental conditions at the transcript level at both T4 and T24, whereas nucleotide probes specific for this gene are available on the microarrays used for the transcriptome analyses. Indeed IL8 is a chemokine known to act as one of the major mediators of the inflammatory response and has been reported to be up-regulated after LPS stimulation in many mammals including cattle [34,36] and pig [37,18]. As opposed to other species, the possibility that T4 might be too late for detecting an up-regulation of IL8 in rabbits cannot be excluded. Recent reports have shown that bacterial-host interactions affect histone acetylation, phosphorylation and methylation state at the TLR4 and IL8 promoter in host cells [38,39]. In cattle, it has been hypothesized that host individual variability in response to LPS stimulation could be due to epigenetic variations modulating individual IL8 expression in PBMCs [34] and in dermal fibroblasts [36].

In addition, DEFB1 and BPI were down-regulated at T24 compared to mock-stimulation, whereas they were expected to be up-stimulated due to their bactericidal activity [40-42]. Defensins form a family of microbicidal and cytotoxic peptides, and DEFB1 encodes an antimicrobial peptide implicated in the resistance of epithelial surfaces to microbial colonization [43]. Beta defensins have thus an antimicrobial activity that defend epithelial surfaces including the skin, gastrointestinal, and respiratory tracts. BPI encodes a LPS binding protein associated with neutrophil granules in humans, and has bactericidal activity on gram-negative organisms [44]. Upon LPS activation, BPI may have several functions, as inhibition of endothelial cell growth and inhibition of dendritic cell maturation, or as an anti-angiogenic, chemoattractant or opsonization agent [45]. To our knowledge, no study has shown down-regulation of DEFB1 or BPI in rabbit PBMCs upon LPS activation. However, a down-regulated expression of DEFB1 has been shown following infections by several pathogens (*Shigella* and *Cryptosporidium parvum*) in epithelial cells of the digestive and respiratory tracts in humans or mice [46-48]. In our experimental model, we do not have a clear explanation for the down-regulation of DEFB1 and BPI, but our results suggest the onset of an immune escape mechanism upon LPS stimulation that needs further investigation. We cannot rule out that both genes are induced early after stimulation, before the time window chosen for this study. Additional data are required to better assess why no increase of IL8 transcription was detected during the global inflammatory process induced by LPS stimulation in rabbit PBMCs between 4 and 24 hours post stimulation, and why a down-regulation of bactericidal activity-related genes was found at T24. It could be interesting to carry out new kinetics studies that include earlier time points (e.g. one hour post-stimulation), as well as a mid-time point (e.g. 12 hours post stimulation) and a late time point at 48 hours post-stimulation, thus providing new data to validate or not the hypothesis that rabbits differ from mice and humans for the *in vitro* production of IL8, and up-regulation of DEFB1 and BPI by PBMCs upon LPS stimulation.

### Sets of genes commonly affected by LPS stimulation in various mammalian species

Our results have confirmed a wide range of genes, other than cytokines and chemokines, which were affected by LPS stimulation in rabbit PBMCs. This has already been reported in other species, thus illustrating shared innate immunity responses among mammals.

The SAA1 gene belongs to the family encoding the serum amyloid A (SAA) proteins. As reported in pigs [18], the SAA1 gene was found up-regulated upon LPS stimulation in PBMCs at T24. The association of increased levels of SAA proteins with inflammatory states has been largely reported [49], but the precise functions of SAA proteins still need clarification. SAA has been reported to be involved in the establishment and maintenance of inflammation, and as an opsonin it facilitates phagocytosis of Gram-negative bacteria [50]. SAA acts as an antiapoptotic agent for neutrophils [51], and directly drives a regulatory program in neutrophils by stimulating them to produce IL10 that has anti-inflammatory effects [52]. The strong up-regulation of IL10 detected in rabbit PBMCs at T24 might be related to this role of SAA. In addition, SAA was recently shown to induce mitogenic signals in regulatory T cells, by enhancing the secretion of IL1B and IL6 by monocytes, and driving them toward a phenotype resembling immature dendritic cells [53]. Indeed, IL6 and IL1B genes were both strongly up-regulated together with SAA1. Our results suggest that the rabbit is an interesting species for further study of SAA-associated regulatory mechanisms, which could be shared across mammals.

The S100A12 gene was strongly up-regulated in rabbit LPS-stimulated PBMCs at T24, as reported in pig PBMCs under similar LPS stimulation conditions [18]. S100A12 gene belongs to the family that encodes the S100 proteins. Approximately 25 different S100 proteins have been characterized so far [54]. A common feature shared by all the S100 proteins is the presence of a pair of calcium-binding helix-loop-helix domains referred to as EF-hand calcium-binding regions toward either end of the protein and separated by a hinge region [55]. The broad diversity of S100 proteins is related to a multitude of biological functions such as cell growth, cell differentiation, and cell survival in numerous physiological and pathological conditions in all cells of the body. Furthermore, all S100 proteins are likely to induce some changes in the actin cytoskeleton organization [54]. S100A12 is expressed in the myeloid cell lineage, and is found in abundance in granulocytes, monocytes, and lymphocytes in human. Extracellular S100A12 can induce directional migration and chemotactic responsiveness of monocytes and neutrophils *in vitro* [54].

At T24 post LPS stimulation, we detected a very strong transcription increase of the gene F3 that encodes the coagulation factor III or thromboplastin, a cell surface glycoprotein that enables cells to initiate the blood coagulation cascades. It has been long reported that blood cells generate thromboplastin activity upon incubation with LPS or Gram-negative bacteria [56]. It has been further established that monocytes are the blood cells that possess this property [57], and that collaboration with lymphocytes is required for a rapid induction of procoagulant activity [58]. Induction of pro-coagulant activity of hepatic macrophages by LPS has already been demonstrated in rabbits [59]. In this paper, we demonstrate by a genome-wide and a non-focused approach that a pro-coagulant activity is also strongly induced by LPS in PBMCs in the rabbit.

A concerted action of S100A12, SAA, and thrombotic functions during inflammatory diseases has been reported in humans [60]. Here, we clearly show that in rabbit PBMCs stimulated *in vitro* by LPS, a strong combined increase in the expression of the corresponding rabbit genes (S100A12, SAA1, F3) is detected.

### PMA-Ionomycin stimulation affects many more genes than LPS stimulation

PMA is a diester of phorbol and a potent tumor promoter often used in biomedical research to activate the signal transduction enzyme protein kinase C (PKC). For *in vitro* cell stimulation, PMA is used in conjunction with ionomycin, a Ca^2+^ ionophore produced by the bacterium *Streptomyces conglobatus*. The number of DE genes after PMA-Ionomycin stimulation was found to be 15 and 20 times higher at T4 and T24, respectively, by comparing to the number of DE genes after LPS stimulation. These findings are in agreement with a similar study in swine in which we reported that ten times more genes were DE after PMA-Ionomycin stimulation than after LPS stimulation [18]. This difference is consistent with the dynamics of the responses to LPS and PMA-Ionomycin, since it was observed at T4 and maintained at T24. As previously discussed [18], this observation might reflect that LPS targets only a limited set of monocytes and macrophages expressing CD14 [61], while PMA-Ionomycin has a much wider spectrum of target cells. We found 515 probes that were down- and up-regulated at T4 and T24, respectively, and vice versa for another 227 probe set. All these genes relate to the same biological functions than those associated to genes found only up- or down-regulated at both T4 and T24, thus likely reflecting a dynamic process that includes combined actions of genes toward a concerted immune response.

### PMA-Ionomycin stimulation induced early Th2, iTreg (induced Tregs), and Th17 responses at T4 followed by an increase in the Th1 response at T24

At T4, the cytokine profile revealed a very strong up-regulation of IL13 and IL4, which are produced by Th2 population subsets [61]. Both cytokines were still up-regulated at T24 in comparison to mock-stimulated cells but with a much lower fold-change than at T4 (Table 5). We could not detect up-regulation of transcription factors that induce cells to commit to a Th2 phenotype such as STAT6 and GATA3. Similarly, IL10 could be detected at T4 but the up-regulation was decreased at T24, as previously described in pigs [62]. This cytokine is identified as a hallmark of iTreg lymphocytes [61]. The FOXP3 gene known to be involved in iTreg lymphocyte differentiation was not found to be affected by PMA-Ionomycin stimulation. In addition, IL17F was moderately up-regulated at T4 (Table 5) in comparison to mock-stimulated PBMCs, and IL17A was found to be DE at both T4 and T24 with a decrease in the FC between stimulated and non-stimulated PBMCs at T24 in comparison to T4. All these results suggest a very early expression of Th2, iTreg, and Th17-associated cytokines with a progressive slow-down after T4. Conversely, we detected an increase in Th1 phenotypes between T4 and T24. Indeed, the two lineage-defining transcription factors STAT4 and TBX21 (or T-bet) were significantly up-regulated at T4, and at T4 and T24, respectively. STAT4 and TBX21 genes drive the Th1 cell commitment and IFNG is the main Th1 cytokine. These data show that PMA-Ionomycin stimulation induces a Th1 response that is not only maintained, but was found to increase at T24. This coincides with results we have previously reported, where a predominance of a Th1 response after PMA-Ionomycin treatment of PBMCs was seen in swine for T24. Our results in rabbits suggest that the Th1 response induced by PMA-Ionomycin might be maintained on a longer term than other responses, thus confirming a predominance of a Th1 response after 24 h stimulation as previously shown in pigs [18]. Recent reports have demonstrated a plasticity of T-helper cell differentiation [61], thus suggesting that T cell differentiation is not always the result of a direct differentiation from naïve T cells but that new cell commitments may also occur after a reprogramming of differentiated cells [61]. From our data, we cannot determine whether the observed Th1 phenotype increase is due to the reprogramming of differentiated T cells or to the direct differentiation of naïve T cells.

### A strong up-regulation of CSF2 and a down-regulation of IL16 induced by PMA-Ionomycin stimulation in favor of cell proliferation

The most up-regulated gene at T4 and T24 after PMA-Ionomycin stimulation was CSF2, a cytokine that functions as a white blood cell growth factor. CSF2 stimulates stem cells to produce granulocytes (neutrophils, eosinophils, and basophils) and monocytes [63]. By contrast, the cytokine IL16 was down-regulated at both T4 and T24, with a high negative fold-change.

Several studies have shown that IL16 is constitutively expressed in peripheral blood monocytes and that there is a direct link between IL16 expression and apoptosis [64,65], and with autoimmune and allergic diseases [66]. Whereas some genes (including IL10, IL16, and TLR6) have been shown to be down-regulated during Fas-mediated or camptothecin-induced apoptosis in human polymorphonuclear leukocytes [67], our results confirmed previous studies where down-regulation of IL16 is associated with T cell activation and proliferation [68,69]. IL16 has also been described as an important growth-promoting factor in multiple myeloma [70].

## Conclusion

In this report, we have characterized the gene profiling modification of rabbit PBMCs upon *in vitro* stimulation by either LPS or a mixture of PMA and ionomycin, thus providing genome-wide expression data on immune responses for the rabbit species. Our results were consistent with data previously cited for other mammalian species, thus confirming that the rabbit is a relevant model for immunity studies.

Presently, the rabbit is no longer a species lacking genomic tools. The availability of a 7X coverage of the genome sequence [7], together with the progress of related information on annotation and SNP variations, constitute highly valuable tools for launching large-scale genome-wide studies in this species [4]. For transcriptomic studies, it is now widely acknowledged that RNAseq-approaches are well adapted to exhaustively detect all transcription variations between different conditions together with SNP variations among individuals. The rabbit is a very useful model in biomedical research [4,71]. As a livestock species, there is a need for large scale studies on resistance to disease and reduction of the use of antibiotics in production systems. The rabbit species does not have the same financial means as other species such as cattle. This study shows that a commercial well-annotated microarray is now available for rabbits, and can be used in combination with RNAseq approaches. Due to their efficiency in the study of immune responses, as well as their cost-effectiveness, the use of microarrays can be easily expanded to large cohorts of animals either for biomedical research or for improving knowledge on immunity and resistance to diseases to promote safe and efficient farm systems.

## Methods

### Enrichment and annotation steps

The first step was to compare the existing Agilent customized microarray design 8x60K (GPL16482) with the set of genes involved in the pig immune response [18] and then using the Gene Ontology term GO:0002376 (immune system process) on rabbit, pig, mouse and human species. Immunity-related rabbit sequences were retrieved by GeneID and RefSeq search or by analysis for sequence similarity by BLAST. All 60-mer length oligonucleotide probes were designed using eArray (Agilent technologies). All new probes were spotted in triplicates. The platform was registered under the GEO accession number GPL16709.

The second step was the annotation of the probes present on the microarray. Preliminary work based on chromosomal coordinates was made. Then we performed a BLAST search of the rabbit probe sequences versus a new RNAseq annotated database (Broad Institute, Cambridge, MA, USA).

### PBMCs: isolation and stimulation

All experiments involving animals complied with the institutional guidelines of the French Ethical Committee. All animals were bred and slaughtered in an approved animal facility (UCEA, approval number A-78-322-4). Experimental protocols were conducted under the supervision of the animal welfare committee in charge of this animal facility (Protocol registered under number 272). V. Duranthon holds an authorization certificate issued by the French governmental administration to experiment on animals (Authorization number B78-101). PBMCs were obtained from 13 mL of heparinized whole blood of four adult female New Zealand White rabbits (numbered 1, 2, 4, and 5) by density gradient separation using ficoll-histopaque. A total of 5 × 10^5^ cells were suspended into RPMI 1640 medium (BioWhittaker, Belgium) supplemented with 200 mM/L of L-glutamine (Sigma-Aldrich, Saint Louis, MO, USA), 10% heat inactivated fetal bovine serum, 1% penicillin and streptomycin (Life Technologies, Carlsbad, CA, USA). PBMCs were stimulated with 1 μg/mL of LPS (Sigma, France) or a mixture of PMA (Sigma, France) at 10 ng/mL and ionomycin (Sigma, France) at 1 μg/mL. For mock-stimulation, a buffer without stimulation agents was added to the cells similarly to stimulation conditions. Cells were incubated at 37°C with 5% CO_2_ and harvested 4 and 24 hours post-stimulation for further RNA extraction.

### RNA extraction

Total RNA was extracted from PBMCs using the RNeasy Mini Kit (Qiagen, Valencia, CA, USA) and residual contaminating genomic DNA was cleaned by using RNase-free DNase I (Qiagen, Valencia, CA, USA). RNA samples were quantified using a NanoDrop ND-1000 spectrophotometer (Thermo Fisher Scientific, Waltham, MA, USA). The RNA integrity was then assessed on a Bioanalyzer 2100 using RNA 6000 Nano chips (Agilent Technologies, Santa Clara, CA, USA).

### RNA labeling and microarray processing

Transcriptional profiling was performed using the previously described custom array. A total of 24 samples was processed. All steps were performed by the CRB GADIE facility (INRA Jouy-en-Josas, France, http://crb-gadie.inra.fr/). Cyanine-3 (Cy3) labeled cRNA were prepared using 200 ng of total RNA using the One-Color Low Input Quick Amp Labeling kit (Agilent Technologies, Santa Clara, CA, USA) following the recommended protocol. Specific activities and cRNA yields were determined using the NanoDrop ND-1000 (Thermo Fisher Scientific, Waltham, MA, USA).

For each sample, 600 ng of Cy3-labeled cRNA (specific activity > 6.0 pmol Cy3/μg of cRNA) were fragmented at 60°C for 30 minutes in a reaction volume of 25 μl containing 25× Agilent Fragmentation Buffer and 10× Agilent Blocking Agent, following the manufacturer’s instructions. Subsequently, 25 μl of 2× Agilent Hybridization Buffer were added to the fragmentation mixture and hybridized to the Rabbit Custom Gene Expression Microarrays (Agilent Technologies, design ID: 042421) for 17 hours at 65°C in a rotating Agilent hybridization oven (Agilent Technologies). After hybridization, microarrays were washed 1 minute at room temperature with the GE Wash Buffer 1 (Agilent Technologies) and 1 minute at 37°C using the GE Wash Buffer 2 (Agilent Technologies), then dried instantly.

Immediately after washing, the slides were scanned using a G2565CA Scanner System (Agilent Technologies), which used a scan protocol with a resolution of 3 μm and a dynamic range of 20 bits. The resulting .tiff images were analyzed with the Feature Extraction Software v10.7.3.1 (Agilent Technologies), using the GE2_107_Sep09 protocol. The microarray data were submitted to the GEO and received the accession number GSE59263.

### Statistical analysis

Microarray data were analyzed using the R/Bioconductor software package Limma (Linear Models for Microarray Data) [72]. The median signals of all probes were first Log 2-transformed, and then these data were normalized by a median normalization. After averaging the normalized data of the probes targeting the same genes, a linear model was fitted for all probes using lmFit function, with type of stimulation and time as factors. As we had a low number of biological replicates, we also used the empirical Bayes approach to compute moderated t-statistics and log-odds of differential expression. The differential analysis was done with averaged and single probes, and DE genes were identified based on contrasts for the (stimulation × time levels) interactions. The P-values were corrected for multiple testing using a false discovery rate method (q-value < 0.05), which provides an estimate of the fraction of false discoveries among the significant terms. A PCA and a HCA were performed. In the results and discussion, the FC values were transformed into linear values for a better biological understanding. Venn diagrams were produced using the Limma package [72]. Finally, the significant DE genes were analyzed with IPA (Ingenuity Systems, Mountain View, CA; http://www.ingenuity.com) to obtain the top biological functions and relevant networks.

### Real time quantitative reverse transcription PCR

For real-time PCR analyses, 100 ng of DNase I treated total RNA were reverse-transcribed using Superscript III with random hexamers (Life Technologies, Carlsbad, CA, USA). Triplicate reactions were performed in a final volume of 20 μL, mixing 20 μL cDNA, 120 nM primers and 10 μL SYBR Green PCR Master Mix (Life Technologies, Carlsbad, CA, USA), using a QuantStudio 12 K Flex system (Life Technologies, Carlsbad, CA, USA). Primers were defined either using the Primer3 Software (Life Technologies, Carlsbad, CA, USA) or manually designed (Additional file 9). The genes B2M and GAPDH were chosen as housekeeping genes and the 2^-∆∆Ct^ method was used to calculate the fold change in gene expression. For comparison with microarray data, Spearman correlation was calculated between the Log2 (FC) provided by the microarray study and the Log2 (Ct) provided by the RT-qPCR study [73].

## Additional files

Additional file 1:
**Gene set related to the immune response.** The file gene_set_S1.xlsx is an excel file, which contains the set of immune genes missing on the previous array with associated Ensembl ID. Additional file 2:
**Hierarchical clustering. Pearson correlation distance – Ward linkage.** The file hierarchical_clustering_S2.docx is a word file, which contains a hierarchical clustering of samples, using the Pearson correlation distance and ward linkage. Additional file 3:
**List of differentially expressed genes (FDR < 0.05) in rabbit PBMCs after LPS activation by comparison to mock-stimulation at T4 post-stimulation.** The file LPS_T4_S3.xlsx is an excel file, which contains all differentially expressed genes after LPS activation by comparison to mock-stimulation at T4 post-stimulation. Additional file 4:
**List of differentially expressed genes (FDR < 0.05) in rabbit PBMCs after LPS activation by comparison to mock-stimulation at T24 post-stimulation.** The file LPS_T24_S4.xlsx is an excel file, which contains all differentially expressed genes after LPS activation by comparison to mock-stimulation at T24 post-stimulation. Additional file 5:
**List of differentially expressed genes (FDR < 0.05) in rabbit PBMCs after PMA-Ionomycin activation by comparison to mock-stimulation at T4 post-stimulation.** The file PMA_Iono_T4_S5.xlsx is an excel file, which contains all differentially expressed genes after PMA-Ionomycin activation by comparison to mock-stimulation at T4 post-stimulation. Additional file 6:
**List of differentially expressed genes (FDR < 0.05) in rabbit PBMCs after PMA-Ionomycin activation by comparison to mock-stimulation at T24 post-stimulation.** The file PMA_Iono_T24_S6.xlsx is an excel file, which contains all differentially expressed genes after PMA-Ionomycin activation by comparison to mock-stimulation at T24 post-stimulation. Additional file 7:
**MHC profile of PMBCs after PMA-Ionomycin activation at T4 and T24.** The file MHC_profile_S7.docx is a word file, which contains genes of the Major Histocompatibility Complex found differentially expressed at T4 or/and T24. Additional file 8:
**Log2(FC) and Log2(Ct) of Microarray and qRT-PCR data.** The file PCR_validation_S8.docx is a word file, which contains microarray and qRT-PCR data used for the validation of the microarray study. Additional file 9:
**Primer sequences for qRT-PCR validation.** The file primers_sequences_S9.docx is a word file, which contains primer sequences (forward and reverse) of genes used for qRT-PCR validation, with amplicon size and accession number.

## Abbreviations

PBMC: Peripheral blood mononuclear cell
LPS: Lipopolysaccharide
PMA: Phorbol myristate acetate
DE: Differentially expressed
RNA-seq: RNA sequencing
FC: Fold change
FDR: False discovery rate
qRT-PCR: Quantitative Real-Time PCR
