# Supplementary figures and images for: Genome-wide immunity studies in the rabbit: transcriptome variations in peripheral blood mononuclear cells after in vitro stimulation by LPS or PMA-Ionomycin
Source: BMC Genomics. 2015 Jan 23;16(1):26. doi: 10.1186/s12864-015-1218-9 (PMC4326531; doi:10.1186/s12864-015-1218-9)

**Additional file 2:** Hierarchical clustering. Pearson correlation distance – Ward linkage


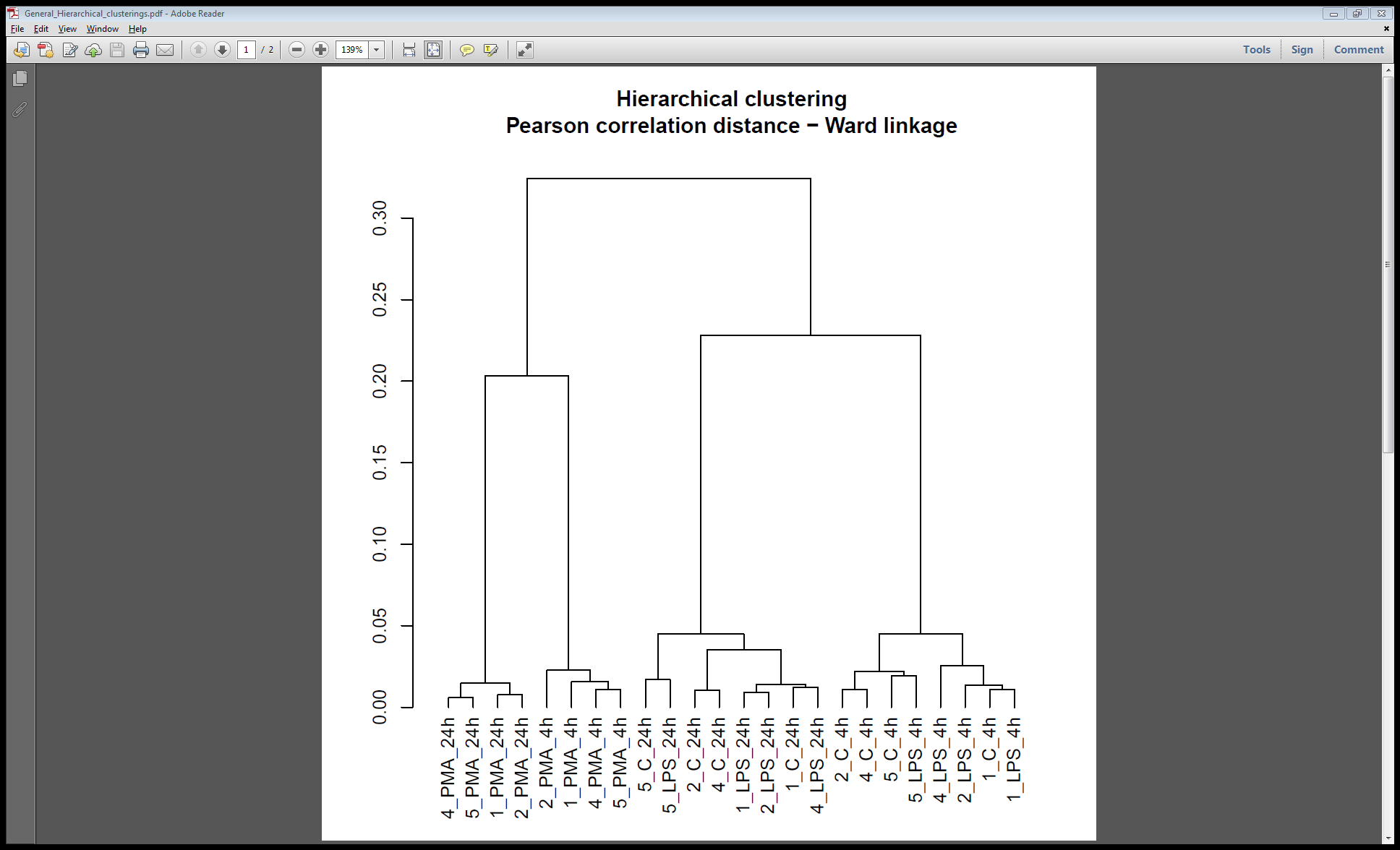

Supplement: Additional file 2: — Hierarchical clustering. Pearson correlation distance – Ward linkage. The file hierarchical_clustering_S2.docx is a word file, which contains a hierarchical clustering of samples, using the Pearson correlation distance and ward linkage. [file 12864_2015_1218_MOESM2_ESM.docx]
