# Supplementary material for: Genome-wide immunity studies in the rabbit: transcriptome variations in peripheral blood mononuclear cells after in vitro stimulation by LPS or PMA-Ionomycin
Source: BMC Genomics. 2015 Jan 23;16(1):26. doi: 10.1186/s12864-015-1218-9 (PMC4326531; doi:10.1186/s12864-015-1218-9)
Supplement: Additional file 7: — MHC profile of PMBCs after PMA-Ionomycin activation at T4 and T24. The file MHC_profile_S7.docx is a word file, which contains genes of the Major Histocompatibility Complex found differentially expressed at T4 or/and T24. [file 12864_2015_1218_MOESM7_ESM.docx]

**Additional file 7:** Transcription profile of genes involved in the MHC class I and class I presentation pathways in PBMCs at T4 and T24 post-stimulation by PMA-Ionomycin.

|  | **ID** | **T4** | | **T24** | |
| --- | --- | --- | --- | --- | --- |
|  |  | **FC**^1^ | **adj.P.Val** | **FC**^1^ | **adj.P.Val** |
| MHC class I  presentation  pathway | HSPD1 | ND^2^ |  | 5.94 | 1.67E-13 |
|  | HSPB11 | ND |  | 5.32 | 2.15E-13 |
|  | HSPH1 | ND |  | 5.14 | 6.43E-13 |
|  | PSMB9 | ND |  | 4.31 | 4.66E-06 |
|  | TAP1 | ND |  | 2.47 | 1.03E-02 |
|  | HSP90AA1 | ND |  | 2.25 | 5.12E-08 |
|  | HSP90AB1 | ND |  | 2.23 | 1.72E-04 |
|  | PSMB2 | ND |  | 2.21 | 1.17E-07 |
|  | PSMB1 | ND |  | 2.19 | 7.35E-09 |
|  | PSMB4 | ND |  | 2.02 | 4.99E-06 |
|  | PSMB5 | ND |  | 1.99 | 6.10E-08 |
|  | HSPA8 | ND |  | 1.82 | 8.84E-03 |
|  | HSPA9 | ND |  | 1.66 | 7.79E-07 |
|  | PSMB3 | ND |  | 1.66 | 3.96E-02 |
|  | CANX | ND |  | 1.59 | 5.60E-04 |
|  | HSPA4 | ND |  | 1.57 | 5.84E-05 |
|  | PSMB7 | ND |  | 1.38 | 4.26E-03 |
|  | HSP90B1 | ND |  | 1.23 | 4.50E-02 |
|  | HSPA13 | ND |  | -1.30 | 1.78E-02 |
|  | HSPBAP1 | ND |  | -1.55 | 2.17E-06 |
| MHC class II  presentation  pathway | HLA-DPB1 | ND |  | -2.19 | 5.96E-05 |
|  | RLA-DQB | ND |  | -6.34 | 4.69E-08 |
|  | HLA-DPA1 | ND |  | -7.83 | 2.12E-03 |
|  | RLA-DMB | -5.75 | 2.65E-10 | -8.42 | 5.76E-12 |
|  | RLA-DQA | ND |  | -12.95 | 1.46E-06 |
|  | HLA-DMA | -4.07 | 1.21E-06 | -16.49 | 1.76E-11 |
|  | HLA-DOA | ND |  | -18.91 | 1.17E-05 |
|  | RLA-DRB1 | -2.58 | 4.74E-02 | -24.13 | 9.33E-08 |
|  | RLA-DR-ALPHA | ND |  | -27.20 | 1.19E-09 |

^1^ FC: fold change

^2^ ND: not detected as differentially expressed
