# Supplementary material for: Genome-wide immunity studies in the rabbit: transcriptome variations in peripheral blood mononuclear cells after in vitro stimulation by LPS or PMA-Ionomycin
Source: BMC Genomics. 2015 Jan 23;16(1):26. doi: 10.1186/s12864-015-1218-9 (PMC4326531; doi:10.1186/s12864-015-1218-9)
Supplement: Additional file 8: — Log2(FC) and Log2(Ct) of Microarray and qRT-PCR data. The file PCR_validation_S8.docx is a word file, which contains microarray and qRT-PCR data used for the validation of the microarray study. [file 12864_2015_1218_MOESM8_ESM.docx]

**Additional file 8:** Log2(FC) and Log2(Ct) of Microarray and qRT-PCR results, respectively

|  | Gene  Name | LPS T4 | | LPS T24 | | PMA-ionomycin T4 | | PMA-ionomycin T24 | |
| --- | --- | --- | --- | --- | --- | --- | --- | --- | --- |
|  |  | Microarray^1^ | qPCR^2^ | Microarray^1^ | qPCR^2^ | Microarray^1^ | qPCR^2^ | Microarray^1^ | qPCR^2^ |
| Ref gene | B2M | *No DE* | *No DE* | *No DE* | *No DE* | *No DE* | *No DE* | *No DE* | *No DE* |
| Ref gene | GAPDH | *No DE* | *No DE* | *No DE* | *No DE* | *No DE* | *No DE* | *No DE* | *No DE* |
| Test gene | IL1B | *No DE* | *No DE* | 4.18 | 4.16 | *No DE* | *No DE* | -3.55 | -2.98 |
| Test gene | IL2 | *No DE* | *No DE* | *No DE* | *No DE* | 5.32 | 10.17 | 4.35 | 8.65 |
| Test gene | IL6 | *No DE* | *No DE* | 4.75 | 5.41 | 2.34 | 1.87 | 2.01 | 1.10 |
| Test gene | IL10 | *No DE* | *No DE* | 4.14 | 4.14 | 5.06 | 4.77 | 2.01 | 2.50 |
| Test gene | IFNG | *No DE* | *No DE* | *No DE* | *No DE* | 3.09 | 7.03 | 3.62 | 6.82 |
| Test gene | TNF | *No DE* | *No DE* | 2.56 | 2.68 | *No DE* | *No DE* | 2.36 | 1.80 |
| Test gene | CCL4 | *No DE* | *No DE* | 3.19 | 3.86 | *No DE* | *No DE* | 2.33 | 1.43 |
| Test gene | CD14 | -1.21 | -1.13 | *No DE* | *No DE* | *No DE* | *No DE* | *No DE* | *No DE* |

^1^ Values are in Log2(Fold Change)

^2^ Values are in Log2(Ct)
