# Supplementary material for: Genome-wide immunity studies in the rabbit: transcriptome variations in peripheral blood mononuclear cells after in vitro stimulation by LPS or PMA-Ionomycin
Source: BMC Genomics. 2015 Jan 23;16(1):26. doi: 10.1186/s12864-015-1218-9 (PMC4326531; doi:10.1186/s12864-015-1218-9)
Supplement: Additional file 9: — Primer sequences for qRT-PCR validation. The file primers_sequences_S9.docx is a word file, which contains primer sequences (forward and reverse) of genes used for qRT-PCR validation, with amplicon size and accession number. [file 12864_2015_1218_MOESM9_ESM.docx]

**Additional file 9:** Primer sequences for qRT-PCR validation

| Gene name (Symbol) | Forward | Reverse | Amplicon size (bp) | Accession number |
| --- | --- | --- | --- | --- |
| B2M | CCCAGAAGTACAGAAAACATGG | TCAAGGAGCCTATGACAGTGAA | 176 | NM_004048 |
| GAPDH | AGTGACACCCACTCCTCCAC | TGTAGCCAAATTCGTTGTCG | 100 | NM_001082253 |
| IL1B | TTGAAGAAGAACCCGTCCTCTG | CTCATACGTGCCAGACAACACC | 128 | NM_001082201 |
| IL2 | TCCAGGATGCTCACATTCAA | GCACTTCCTCCAGAGGTTTG | 100 | NM_001163180 |
| IL6 | GAAAACACCAGGGTCAGCAT | TGAGGGTGGCTTCTTCATTC | 97 | NM_001082064 |
| IL10 | AGAACCACAGTCCAGCCATC | TTTTCACAGGGGAGAAATCG | 106 | NM_001082045 |
| IFNG | TTCAGCACATTGGAGAGTTCA | GCAACCTCACTAAGATGGATGA | 103 | NM_001081991 |
| TNF | CTCTTCTGCCTGCTGCACTT | GGCCACAGGGTTGACTAGAT | 87 | NM_001082263 |
| CCL4 | GAGACCACCAGCCTCTGCTC | TCAGTTCAGTTCCAAGTCATCCAC | 123 | NM_001082196 |
| CD14 | TCACCTAGACCTCAGCCACA | CACCTGCTGCAGTCCAGTAA | 106 | NM_001082195 |
